# Wide-Range Portrayal of AP2/ERF Transcription Factor Family in Maize (*Zea mays* L.) Development and Stress Responses

**DOI:** 10.3390/genes14010194

**Published:** 2023-01-11

**Authors:** Cheng Cheng, Likun An, Fangzhe Li, Wahaj Ahmad, Muhammad Aslam, Muhammad Zia Ul Haq, Yuanxin Yan, Ramala Masood Ahmad

**Affiliations:** 1 State Key Laboratory of Crop Genetics and Germplasm Enhancement, Nanjing Agricultural University, Nanjing 210095, China; 2 College of Agriculture and Forestry Sciences, Qinghai University, Xining 810016, China; 3 Institute of Soil and Environmental Sciences, COMSATS University Islamabad, Abbottabad 22020, Pakistan; 4 Department of Plant Breeding and Genetics, University of Agriculture Faisalabad, Faisalabad 38040, Pakistan; 5 Department of Agronomy, University of Agriculture Faisalabad, Faisalabad 38040, Pakistan

**Keywords:** maize, AP2/ERFs, growth, development, stress responses, phytohormones, qPCR

## Abstract

The *APETALA2*/*Ethylene-Responsive Transcriptional Factors* containing conservative *AP2*/*ERF* domains constituted a plant-specific transcription factor (TF) superfamily, called AP2/ERF. The configuration of the AP2/ERF superfamily in maize has remained unresolved. In this study, we identified the 229 *AP2/ERF* genes in the latest (B73 RefGen_v5) maize reference genome. Phylogenetic classification of the *ZmAP2*/*ERF* family members categorized it into five clades, including 27 *AP2* (*APETALA2*), 5 *RAV* (*Related to ABI3*/*VP*), 89 *DREB* (*dehydration responsive element binding*), 105 *ERF* (*ethylene responsive factors*), and a soloist. The duplication events of the paralogous genes occurred from 1.724–25.855 MYA, a key route to maize evolution. Structural analysis reveals that they have more introns and few exons. The results showed that 32 *ZmAP2/ERFs* regulate biotic stresses, and 24 *ZmAP2/ERFs* are involved in responses towards abiotic stresses. Additionally, the expression analysis showed that DREB family members are involved in plant sex determination. The real-time quantitative expression profiling of *ZmAP2/ERFs* in the leaves of the maize inbred line B73 under ABA, JA, salt, drought, heat, and wounding stress revealed their specific expression patterns. Conclusively, this study unveiled the evolutionary pathway of *ZmAP2/ERFs* and its essential role in stress and developmental processes. The generated information will be useful for stress resilience maize breeding programs.

## 1. Introduction

Dynamic environmental ordeals, including biotic and abiotic stresses, are considered to be vital stimuli affecting the plants’ growth, reproduction, and productivity [1,2]. They have adverse effects on important field crops, such as wheat, rice, and maize, etc. [3] causing a more than 50% reduction in major crop yields worldwide [4]. The growth and developmental processes of maize are subsequently affected by biotic and abiotic factors, such as a scarcity of water, saline stress, and low- and high-temperature stresses that can cause a significant loss in productivity [5,6]. To strive against these environmental stresses, plants have evolved stress-responsive mechanisms, including the quantifiable expression of genes to cope with the stresses at the molecular level [7]. A plant’s response to stress conditions is regulated by the expression of profuse genes working in some fundamental metabolic pathways, i.e., cell metabolism, stress-related proteins, enzymes, secondary metabolites [8], carbohydrates, amino acids, and lipid metabolisms [9,10]. Transcription factors emerged as key regulators in various signaling networks, playing a significant role by improving the growth and development of plants under stress conditions. Transcription factors contain DNA-binding domains that adhere to specific sequences of DNA beside the gene that they control [11,12]. They are categorized into 50–60 families, depending on their amino acid sequences and conserved domains. Many transcription factor families have been studied in maize, i.e., the MADS-box [13], DOF [14], MYB [15], HSP [16], bZIP [17], and NAC [18].

The transcription factor family *AP2/ERF* regulates several regulatory processes, such as the plant’s growth and development, fruit maturity, protection system, metabolism-responsive genes in the signaling pathways of ethylene, and biosynthesis pathways of phytohormones, i.e., ET, CK, GA, JA, ABA in plants [19]. Initially, AP2/ERF TF’s domain was recognized in Arabidopsis and tobacco. The LcERF080 encodes an AP2/ERF protein, which was strongly induced by salt, ABA, MeJA, and SA stresses [20]. Up till now, the investigation and characterization of the AP2/ERF TF family have been explained well in several plants, including *Arabidopsis thaliana* [21], *Oryza sativa* [22], *Brassica oleracea* [23], *Brassica rapa* [24], *Pyrus* [25], *Sesamum indicum* [26], and *Sorghum bicolor* [27].

Generally, *AP2/ERF* TF’s mediated genes undergo downstream by adhering to the GCC-box or DREB elements in the gene’s promoter region and regulate the agronomic traits, e.g., the plant’s growth and development, protection responses, and fruit maturity [28]. Two regions, YRG and RAYD, are present in *AP2/ERF* domain, comprising about 20 and 40 amino acids at the N-terminal region, respectively. The *AP2/ERFs* include the following subfamilies: Apetala 2 (AP2*)*, dehydration-responsive element-binding proteins (DREB), relation to abscisic acid-insensitive 3/ivviparous 1 (RAV), ethylene-responsive factors (ERF), and soloist. The AP2 family contains two repetitive *AP2/ERF* domains or lacks a conserved WLG motif in its domain. The AP2 family mainly regulates the plant’s growth, floral development, leaf shape, and seed growth. The ERF and DREB subfamilies comprise the solo *AP2/ERF* domain [29]. Ethylene response factors (ERFs) were found to be involved in metabolic regulations and might contribute to chromosomal duplication, tandem gene duplication, and transposition in plants [21]. The DREB subfamily of *AP2/ERFs* binds to cis-acting sequences of DRE or the CRT in the promoter region of drought and salt-responsive genes. The *acbf2* mutant in Arabidopsis and overexpressed *OsDREB2A* and *OsDREB1F* mutants in rice result in water scarcity and high salt stress tolerance. The *DREB1* and *DREB2* genes with abscisic acid are well-preserved in monocot and dicot, and perform a significant role in the plant’s abiotic stress responses. The subfamily *RAV* contains a solo *AP2/ERF* domain, can regulate leaf senescence, and takes part in different stress responses [30]. Soloists contain a definite B3 domain.

In recent research work, transcriptomics data have been used to find out the signaling pathways and elements which take part in the plant’s metabolic processes. Next-generation sequencing technology offers insight into both model and non-model plants, reveals the detection of unique genes, alternative splicing, and different transcript evidence, and discovers the SNPs without the availability of gene annotations [31]. The vast extent of the studies of the AP2/ERF TF in *A. thaliana* [21,31] *Populus trichocarpa* [32], *Glycine max* [33], *O. sativa* [34], *Vitis vinifera* [32,35], *Cucumis sativus* [36], *Hevea brasiliensis* [22], *Ricinus communis* [23], *Brassica rapa* [24], *Setaria italica* [25], and *Eucalyptus grandis* [35] provides an improved understanding of this superfamily. This is a highly conserved transcription factor family in plants, though the total number of factors and functional groups can vary between the species due to evolutionary processes [26].

To increase insight into the *AP2/ERFs* family of maize, the in-silico analysis and their expression profiling were performed by using computational tools and qPCR. The phylogenetic analysis, protein motif analysis, chromosomal location, etc. of *ZmAP2/ERFs* members have been expounded. Moreover, by using transcriptomic data, we determined the quantitative expression of *ZmAP2/ERFs* under multiple stress conditions and in various maize tissues. The classification and identification of putative motifs are useful for determining the biological function of *ZmAP2/ERFs*. Further scrutiny has identified candidate factors to be used for the transformation to get stress-resistant maize germplasm. In addition, qPCR allows the investigators to validate the transcriptomic results.

## 2. Materials and Methods

### 2.1. Classification of ZmAP2/ERF Family Members

The maize genome database (B73_RefGen_v5) was obtained from Gramene (http://ensembl.gramene.org/Zea_mays/Info/Index, accessed on 15 June 2022). The Hidden Markov Model (HMM) file corresponding to the AP2 domain (PF00847) and ERF superfamily (PF04404) [37] was downloaded from Pfam (http://pfam.sanger.ac.uk/ accessed on 15 June 2022). The amino acid sequence of the AP2/ERF domain was used as a query sequence to explore the databases using BLASTP. Position-specific BLAST was also used to boost the extent of the database results. The maize AP2/ERF database was also mined from PlantTFDB (http://planttfdb.gao-lab.org/, accessed on 16 June 2022). MaizeGDB and Gramene databases were searched to identify the AP2-like genes. Sequences of all identified members were studied to verify the existence of the conserved AP2 domain by using SMART (http://smart.embl-heidelberg.de/, accessed on 17 June 2022) [38,39]. Briefly, the protein sequences having two AP2 domains were categorized in the same family, named AP2 subfamily, while protein sequences having one AP2 domain were considered to comprise three subfamilies ERF, DREB, and soloist. These three families have a slight difference in their amino acid sequence. Protein sequences sharing one AP2 and one B3 domain were grouped into the RAV family. The genomic, coding, and putative protein sequences of 229 AP2/ERF were obtained from Gramene.

### 2.2. Sequence and Phylogenetic Analysis of ZmAP2/ERF Proteins

The ClustalW program was used to obtain multi-sequence alignment. Phylogenetic trees were created with the allied ZmAP2/ERF protein sequences with MEGAX software by using the neighbor-joining (NJ) method with bootstrap (1000 repeats), Poisson correction, and pairwise deletion [37].

### 2.3. Chromosomal Localization, Duplication, and Conserved Motif Analysis of ZmAP2/ERFs

The physical positions of *ZmAP2*/*ERF*s on chromosomes were obtained from maize genome annotation (*Zea_mays*. B73_RefGen_v5) and mapped to maize chromosomes by using Circos v0.52 [40]. The location of the 229 *ZmAP2/ERFs* on 10 maize chromosomes was visualized by using MapChart 2.32 [40]. To calculate non-synonymous (ka) and synonymous (ks) substitution of each duplicated *ZmAP2/ERF,* the KaKs_calculator 2.0 [41] was used. To search homologous gene pairs among maize, rice, sorghum, and Arabidopsis, BLASTP was performed. Multiple Collinearity Scan toolkit (MCScanX) was implemented to investigate the duplication events, with the default parameters [42]. The intron and exon organization were analyzed by using the TBtools 0.665 [43]. The motif analysis of *ZmAP2*/*ERFs* was conducted by using (MEME: http://meme-suite.org/, accessed on 17 July 2022) [44].

### 2.4. ZmAP2/ERFs Expression Profiling by RNA-seq Data

Expression quantification of all *ZmAP2*/*ERFs* in maize plant [45], under abiotic stresses [45], wounding and oral secretions (OS) to wounds [46], O.F insect attack and JA stresses [47] were obtained from the transcriptomic data that were downloaded from NCBI’s database (accession number GSE50191, PRJNA335771, PRJNA380272, PRJNA299127). Obtained transcriptomic reads were mapped to the maize genome as reference (B73_RefGen_v5) and it was analyzed by using HISAT2 (v2.0.5) [46]. The number of reads were counted by HTSeq (v0.7.1) [47]. The hierarchical clustering of *ZmAP2*/*ERF* genes was created using average linkage with Euclidean distance method by using R software to visualize the expression profile in eight maize plant tissues based on the log10 (FPKM + 1) values of *ZmAP2*/*ERFs*, shown by heat map.

### 2.5. Experimental Material and Treatments

Maize inbred line B73 was grown up to the seedling stage in pots (10.0 cm × 10.0 cm) under controlled conditions: 27/23 °C, light/dark cycle 14/10 h, light density of 250–300 mmolm^−2^ s^−1^ following completely randomized design (CRD). At the V3 stage, plants were subjected to environmental stresses. For salt stress, plants were treated with 200 mM NaCl; after 2h, tissues were collected. For drought stress, the 6-day-old maize seedlings were grown without irrigation until the V3 stage. For heat and cold stresses, seedlings were subjected to 42 °C and 4 °C for 2 h, respectively. Wounding was applied to leaves and samples were collected after 1.5 and 6 h. Samples were collected and instantly stored in liquid nitrogen.

### 2.6. Expression Quantification by qRT-PCR

Total RNA was extracted by using TRIzol reagent (Invitrogen, CA, USA) and cDNA was synthesized by using a PrimeScript 1st strand cDNA Synthesis Kit (TaKaRa, Okinawa, Japan). Gene-specific primers (Supplementary Table S13) were designed by using QuantPrime3. The relative expression of the genes was calculated by using the Ct method, and the qRT-PCR was carried out using a real-time detection system (Roche Diagnostics, Schlieren, Switzerland). Each reaction contained cDNA, 2X SYBR Premix Ex Taq (TaKaRa, Japan), and primers as per recipe [12].

## 3. Results

### 3.1. Phylogenetic Classification of ZmAP2/ERFs Family Members

A methodical approach was carried out to find the *ZmAP2/ERF* subfamily members by using the publicly available genome datasets. The AP2 and ERF keywords were used as queries to find the newest version of the maize genome (V5) in the MaizeGDB and Gramene. Then, BLAST searches were performed by using all of the AP2/ERF sequences to re-examine the acquired sequences. Initially, 236 presumed Maize *AP2/ERF* members were identified. Further verification of this family led to the deletion of seven false-positive sequences. Finally, a total of 229 *ZmAP2/ERF* members were identified and grouped into four subfamilies (Table S1). Each *ZmAP2/ERF* member was specified by the *AP2/ERF* family name based on the standards determined by the Gene Nomenclature Committee (AGNC) [23] then labeled following the Maize nomenclature (Table S1). Nine erroneously predicted *AP2/ERF*s models were manually curated. Different traits, i.e., the length of the coding sequence, the chromosomal location, number of exons, number of introns, *ZmAP2/ERF* domain, subfamily characterizations, variant transcripts, name, and descriptions, are presented in (Table S1).

**Figure 1:**
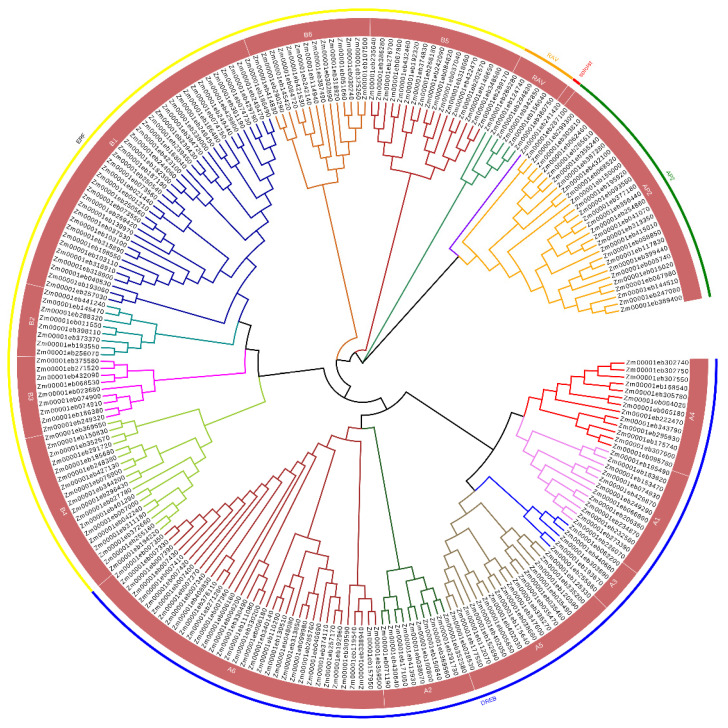
The phylogenetic analysis of *ZmAP2/ERFs*. The different colors are indicating different groups of the *ZmAP2*/*ERF* family. The green color is showing AP2 group of genes, the red color is indicating soloist, the orange color is indicating the RAV group of genes, the yellow color is showing ERF group, and the blue color is showing DREB family factors.

*ZmAP2/ERFs* were categorized into five subfamilies: 89 DREB, 105 ERF, 27 AP2, and five RAV members, along with one soloist (Figure 1). The RAVs and soloist members are shown adjacent to the AP2 transcription factor family. A phylogenetic tree was constructed to explore the evolutionary relationship between the *AP2/ERF* transcription family members in maize. The phylogenetic tree was constructed in the neighbor-joining method by using the full amino acid sequences of ZmAP2/ERF proteins. Resultantly, the dendrogram demonstrates that the *ZmAP2/ERFs* were grouped into five distinct families shown in (Figure 1). The phylogenetic tree (un-rooted) breaks up the *ZmAP2/ERFs* family into groups based on the conservation of group-specific domains among the proteins. Six groups (A1-A6 and B1-B6) have been identified in the DREB and ERF subfamilies, respectively. *Zm00001eb241420* is the only member of the AP2/ERF family which is different from the other family members and categorized as a soloist (Figure 1). The phylogenetic analysis of the *AP2/ERF* superfamily in maize indicated that it has the greatest number of members. The *AP2/ERF* is the major transcription factor family in plants, with 147 members in Arabidopsis [21], 170 members in rice [38,48], 288 members in sunflower [39,49], and 380 members in the soybean genome [40,50].

### 3.2. Conserved Motif Analysis and Intron-Exon Organization in ZmAP2/ERF TFs

**Figure 2:**
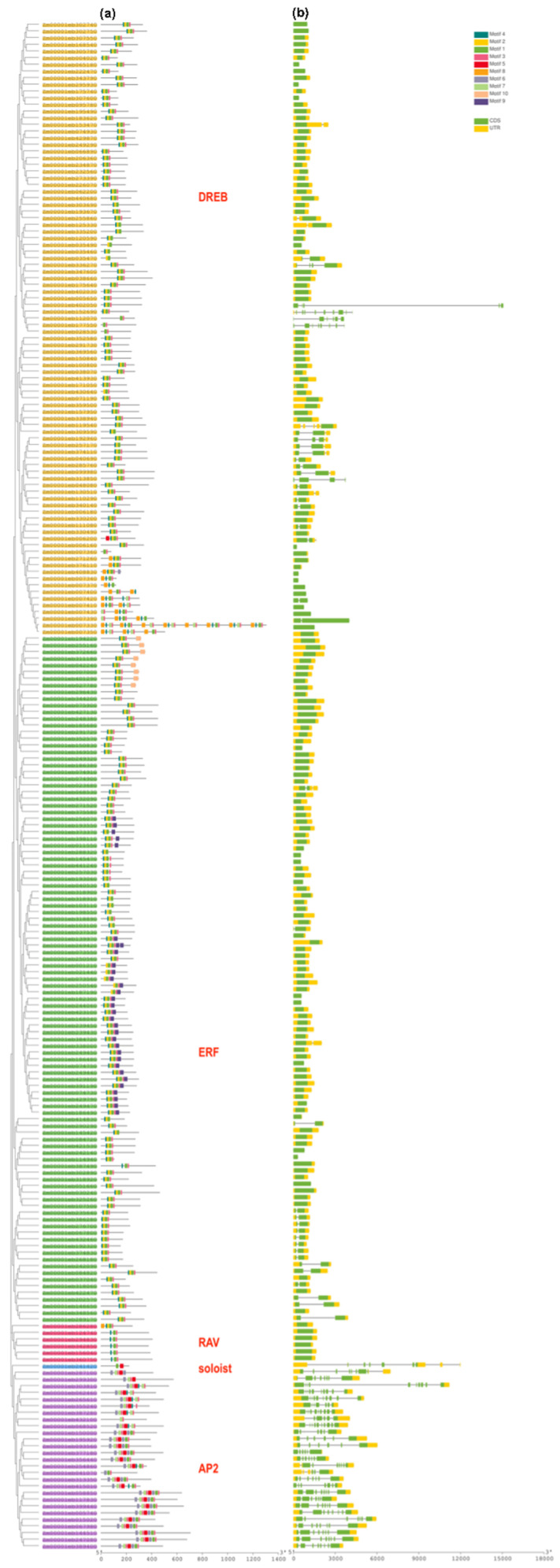
Conserved motif analysis and intron-exon organization of *ZmAP2*/*ERFs.* (**a**) Conserved motifs of different lengths are depicted on the protein map. (**b**) Intron-exon organization of *ZmAP2/ERFs.* At the base of the figure, relative position is displayed at (kilobase) scale.

To obtain further information, the identified amino acid sequences of the maize *AP2/ERF* members were advanced to a conserved motif analysis by using Multiple Em for Motif Elicitation (MEME). A total of ten conserved motifs were found in putative *ZmAP2/ERF* proteins, and they were named motif 1–10 based on the individual’s E-value (Figure 2c and Figure S1). On the N-terminal, motif 7 was the most recurrent, which was found in 48 out of the 53 *ZmAP2/ERF* members, and motif 10 was the second most common motif at this terminal. The results show that, within the same family, the proteins carrying the same conserved motifs were highly similar in function to each other. Some motifs were absent among certain families; for example, the *ZmAP2* family has motif 5, but the *ZmERF* family does not have motif 5 (Figure S1). These results reflect the functional divergence in *ZmAP2/ERF* members. In addition to this, their LOGOS were obtained by the server MEME (Figure S1). Hence, these results suggested that most *ZmAP2/ERF* members carry exceptional features due to differences in their amino acid sequences. These conserved motifs take part in transcriptional activities, protein-protein interactions, DNA interactions, or the structural conformation of proteins.

To expand vision into the evolution of *ZmAP2/ERF* TFs, their coding and genomic sequences were compared to determine the exon-intron organizations (Figure 2b). The *ZmAP2/ERF* TFs’ structure was analyzed via the GSDS online suite to obtain more information regarding their conservation and diversification. The number of exons and introns in *ZmAP2/ERF* members range from 1–10 and 1–4, respectively. The *ZmAP2* family members contain 6, 8, 9, or 10 exons, and the majority of *ZmAP2/ERFs* significantly share a highly conserved structure within the same family or subfamily. Members of a group generally have alike structures, such as how *ZmERFs* have two exons and two introns. Collectively, the conserved motif configurations and structural similarity of *ZmAP2/ERFs* strongly support the consistency of the group classifications.

### 3.3. Chromosomal Arrangement, Paralogous Gene Identification, and Synteny Analysis of the ZmAP2/ERF Transcription Factor Family

The localization of the predicted *ZmAP2/ERFs* was illustrated using the Map Chart software on their corresponding chromosomes in maize (Figure S2). The analysis indicates that the 229 *ZmAP2*/*ERFs* were randomly located across 1–10 chromosomes, and their distribution is (38, 26, 16, 26, 24, 21, 21, 18, 20, and 17 genes, respectively). Chromosome 1 possesses thirty-eight *ZmAP2/ERFs* members and the remaining nine chromosomes carry from sixteen to twenty-six *ZmAP2/ERFs*. Localization revealed that about 78% of the *ZmAP2*/*ERFs* were positioned on chromosomal arms in the maize genome (Figure S2).

**Figure 3:**
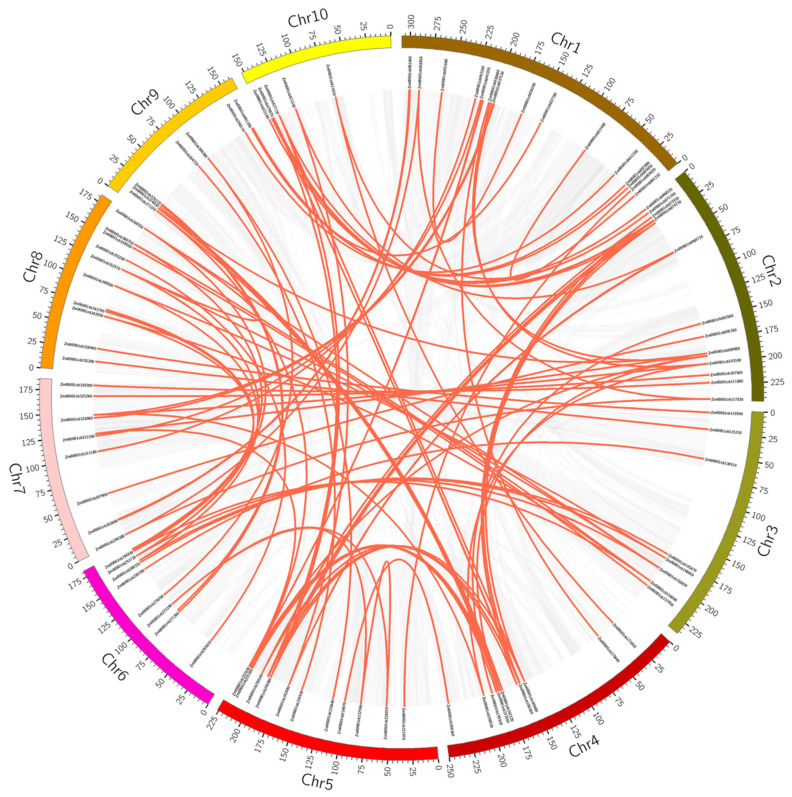
Segmental duplication of *ZmAP2/ERFs* using circos. The red lines show duplicated *ZmAP2/ERFs*. Grey lines show the background genome and chromosome number is designated at the nethermost.

The two important events of transcription factor gene family expansion are segmental and tandem duplications. The evolution and expansion of TFs are usually linked with the duplication of the genome, i.e., segmental or tandem duplications [51]. Segmental duplication results in a discrete occurrence of gene family members on different chromosomes [52]. Tandem amplification results in groups of duplicated genes on the same chromosome [53]. The segmental duplication of maize *AP2/ERFs* using circos is shown in (Figure 3).

The *ZmAP2/ERFs* were unequally dispersed on the 10 Maize linkage groups (LG), as showed by chromosomal lineage analysis in (Figure 3) (Table S2). As per the illustrations of Holub [54], the occurrence of two or more genes within 200 kb of a chromosomal region is termed as a tandem duplication event. On maize linkage groups 5, 6, 7, 8, 9, and 10, *ZmAP2/ERF* genes were grouped into 6 tandem duplication event regions. The analysis presented that several *ZmAP2*/*ERFs* were generated through duplicate events that played a key role in their evolution (Figure 3). The analysis reflects that, during evolution, they are likely to undergo an intense significant selection.

The divergence of maize *ZmAP2*/*ERF* genes is calculated using the Ka and Ks rate per site per year. The non-synonymous (Ka) and synonymous (Ks) substitutions and Ka/Ks rates per site per year for maize genes have been calculated (Table S3). The non-synonymous/synonymous value can estimate the selective pressure on duplicated genes, i.e., it can indicate neutral selection and can find out the selective pressure for replicating genes. The estimated time of duplication was calculated by using the formula T = Ks/2λ. The majority of the segmentally duplicated *ZmAP2*/*ERF* gene pairs showed a Ka/Ks ratio >1). It revealed that non-synonymous changes are more common than synonymous changes in *ZmAP2*/*ERFs*. A Ka/Ks ratio > 1 indicates positive selection, a Ka/Ks ratio = 1 indicates neutral selection, and a Ka/Ks ratio < 1 shows purifying selection. The analysis showed that the lowest value is among the *Zm00001d009103*-*Zm00001d019744* pair (Ka/Ks value = 0.88). It explained that duplications of the paralogous genes in maize occurred from 1.724 to 25.855 MYA. The duplication of *Zm00001d001907* and *Zm00001d026563* occurred very recently: their divergence time is 1.724 MYA.

**Figure 4:**
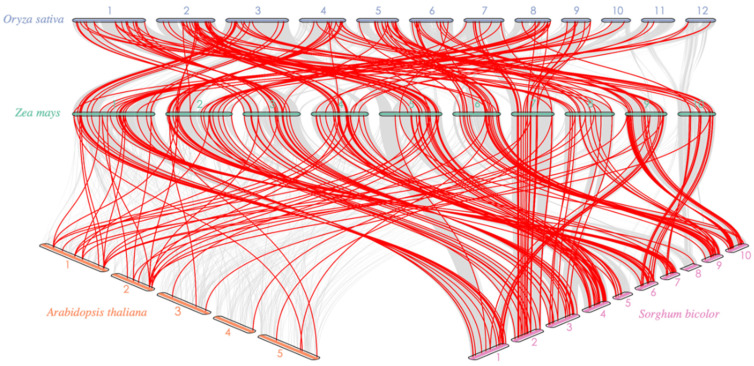
Synteny analysis of *AP2*/*ERFs* between maize, Arabidopsis, rice, and sorghum.

Comparative syntenic map of maize with Arabidopsis, rice, and sorghum was created to advance the insight into the evolutionary mechanism of the *ZmAP2*/*ERFs* (Figure 4). Total 35 *AtAP2*/*ERFs* presented a syntenic linkage to Arabidopsis, 153 in rice and 187 in sorghum (Table S4). The collinear pairing of *ZmAP2/ERFs* was greater with sorghum and rice than with Arabidopsis. In maize, chromosome number five shows no synteny relationship with *A. thaliana*. The analysis depicted that maize, sorghum, and rice (monocots) share a large number of collinear gene pairs as compared to dicots. The Ka/Ks values can be used to calculate the selection pressure for duplicating genes.

### 3.4. Promoter Region Analysis of Maize AP2/ERFs

The cis-acting regulatory elements of the promoter region are directed to the expression associated with different stresses. To study the regulatory functions, we examined the cis-acting elements in the promoter region of all *ZmAP2/ERFs* by using the Plant CARE database. The 1500 kb upstream region of the start codon (ATG) was used to analyze cis-acting regulatory elements of *ZmAP2/ERFs*. The analysis was carried out to find various types of cis-regulatory elements as shown in (Figure S3A).

Briefly, the majority of the *ZmAP2/ERFs* are involved in signal transduction pathways, i.e., the phytohormonal signaling pathway, stress-related pathways, and other regulatory elements. The following analysis illustrated that 28.41% of the *ZmAP2/ERFs* were responsive to phytohormones, followed by 27.38% of *ZmAP2/ERFs* being responsive to biotic and abiotic stresses, while other observed key regulatory elements were reactive to light regulations, defense-related actions, and the binding of proteins. The results indicated that *ZmAP2/ERFs* have varied functions and are involved in many biotic-abiotic and phytohormonal signal transductions (Figure S3B).

The (Figure S3A) presents many cis-elements existing in the promoter region of *ZmAP2/ERFs* (G-box, ARE, GC-motif, I-box, O2-site, TATA-box, LS7, ATC, and GATT). The expression of *ZmAP2/ERFs* may be triggered by several plant hormones, i.e., SA, ET, JA, ABA, GA, and auxin. In addition, the presence of biotic and abiotic stress expression elements (as GC-motif, LTR, WUN-motif, MBS11, MbS1, TC-Rich repeats) in several *ZmAP2/ERFs* promoter regions indicated that these might involve different growth and stress regulations, i.e., heat, cold, salt, and drought, governing the development of the endosperm and flavonoid biosynthesis.

### 3.5. Expression Analysis of ZmAP2/ERFs in Maize Tissues

To explore the mechanism of *ZmAP2*/*ERFs* in maize, their expression pattern was analyzed in different tissues and developmental gradients of maize, including unpollinated silk, female-spikelet, primary roots, vegetative meristem, internode, germinated embryo, endosperm, leaf, and pollen at different developmental stages. The RNA-Seq Atlas of maize offers high-resolution expression data in nine different tissue samples. Publicly available RNA-seq data on NCBI were used to analyze their expression (Tables S5 and S6). It is reported that, among plants, the *AP2*/*ERF* family plays a significant role in the developmental processes [28].

*ZmERF85*, *ZmERF82,* and *ZmDREB5* were highly regulated in pericarp tissues 18 days after pollination. After 24 days of pollination, *ZmDREB69* was overexpressed in the embryo tissues. *ZmERF22* overexpressed in the embryo after 16 days of pollination. Among whole-seed-24DAP, *ZmERF44* was upregulated after 24 days of pollination. *ZmERF23* was overexpressed among endosperm tissues after 16 days of pollination. *ZmERF8* and *ZmERF45* were overexpressed in silk R1. In immature cob, at the V18 stage, *ZmERF64* was overexpressed. *ZmDREB60*, *ZmDREB63*, and *ZmDREB64* were overexpressed in anthers_R1. *ZmDREB81* and *ZmDREB28* were overexpressed among the internode before pollination. *ZmERF10* was overexpressed in the eighth leaf at the V9 stage. *ZmERF90* was overexpressed in the thirteenth leaf. Among immature leave_V9, *ZmDREB74* and *ZmDREB55* were overexpressed. *ZmAP2_4* was overexpressed in pooled_leaves_V1. Among primary root_Z4_7DAS, *ZmDREB17*, *ZmDREB18*, and *ZmDREB45* were overexpressed. Among primary root_GH_6DAS, *ZmDREB9*, *ZmDREB32,* and *ZmDREB84* were highly expressed. Among root_CP_3DAS, ZmDREB16 was overexpressed. Among root cortex tissues, the *ZmERFs* and *ZmDREBs* were overexpressed. *ZmDREB48*, *ZmDREB6,* and *ZmDREB58* were highly expressed in root elongation zone tissues at the five-day stage. (Figure S4A,B)

The maximum number of *ZmAP2* genes was overexpressed in pollen. The *ZmAP2* genes were mostly overexpressed in vegetative meristem tissues, such as *ZmAP2-22*, *ZmAP2-21,* and *ZmAP2-7*. *ZmAP2-21* and *ZmAP2-2* were upregulated among the primary roots. The *ZmRAV*s were overexpressed in pollen tissues, i.e., *ZmRAV2*. *ZmRAV5* and *ZmRAV1* were overexpressed in the vegetative meristem, and *ZmRAV3* was highly regulated in germinated embryos. The soloist was overexpressed in the leaf tissues. (Figure S4B).

### 3.6. Expression Profiling of ZmAP2/ERFs in Response to Biotic and Abiotic Stresses

The advanced study has put forward the observation that *AP2*/*ERFs* play an important role in plant growth and development. The maize *AP2*/*ERF* expression analysis was analyzed for their response to biotic, abiotic, and phytohormonal applications. The *ZmAP2*/*ERFs’* expression under salt, drought, heat, cold, and ABA was analyzed using publicly available maize transcriptomic data [55]. Expression quantification of *ZmAP2*/*ERFs* was also performed under different conditions, i.e., wounding, OA, OF, and JA treatment, by using publicly available maize transcriptomic data [56,57].

**Figure 5:**
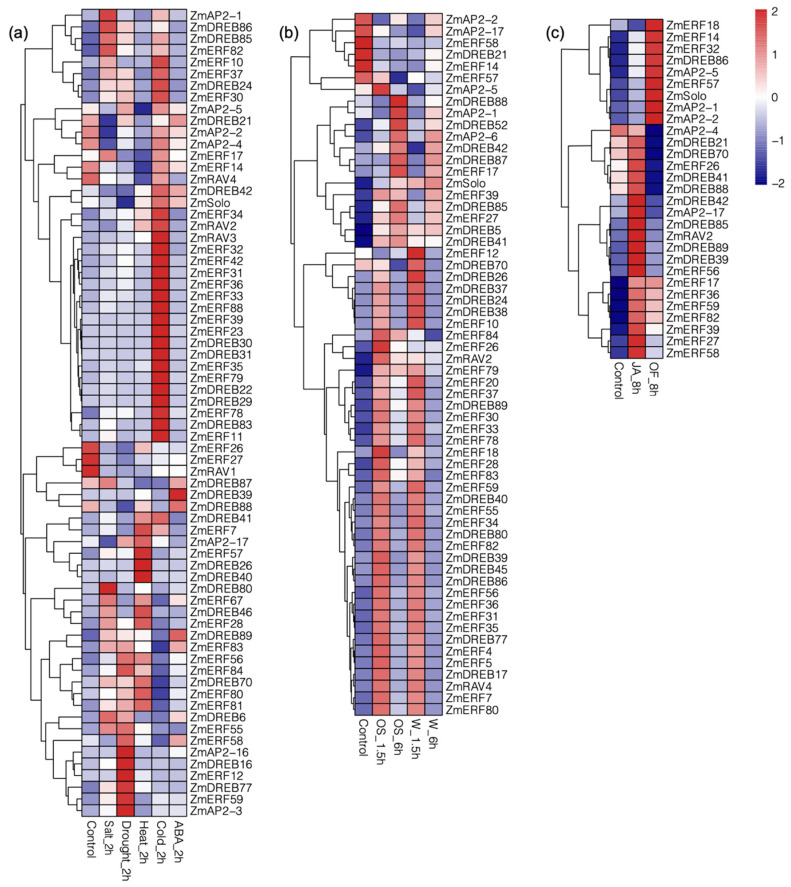
Expression quantification of *ZmAP2*/*ERFs* under abiotic, biotic, and phytohormone treatment. (**a**) Expression analysis of *ZmAP2*/*ERFs* in the response to drought, heat, cold, and ABA treatments. (**b**) *ZmAP2*/*ERF* gene expression in response to wounding and OS treatment shown by heat map. (**c**) Heat map showing expression of *ZmAP2*/*ERF* genes with JA and OF treatments.

The FPKM values of the 229 *ZmAP2/ERF*s (Table S7) were retrieved from transcriptomic data of the maize leaves. The criteria of fold change ≥ 2 and FDR < 0.01 were used to find maize *AP2*/*ERFs* that were differentially expressed under stress and control conditions (Tables S7 and S10). The expression profiling of highly responsive *AP2*, *ERF*, *RAV*, *DREB,* and soloist with an average FPKM value of >10 is shown in (Figure 5). *ZmAP2-1*, *ZmDREB86*, *ZmDREB85*, *ZmERF82,* and *ZmDREB80* were highly expressed under Salt_2h stress. Under Drought_2h conditions, *ZmAP2-3*, *ZmAP2-16*, *ZmERF59*, *ZmERF12*, *ZmDREB77,* and *ZmDREB16* were highly regulated due to water scarcity. Under Heat_2h stress, *ZmERF7*, *ZmERF57*, *ZmDREB26,* and *ZmDREB40* were overexpressed. A large number of genes increased their expression level under low-temperature stress. *ZmERF34*, *ZmERF42*, *ZmERF31*, *ZmERF36*, *ZmERF33*, *ZmERF88*, *ZmERF39*, *ZmERF23*, *ZmERF35*, *ZmERF79*, *ZmERF78*, *ZmERF11*, *ZmERF26,* and *ZmERF27* were highly expressed under the Cold_2h condition. *ZmDREB30*, *ZmDREB31*, *ZmDREB22*, *ZmDREB29*, *ZmDREB83, ZmRAV2,* and *ZmRAV3* were highly expressed under low-temperature stress. *ZmDREBs* only overexpressed in response to ABA_2h treatment. *Zmsoloist* was overly expressed under ABA_2h and cold_2h stress treatments.

In the O.S (oral secretions) from *Mythimna separata* insects and wounding treatment, both the ERF and DREB gene families were highly responsive. Wounding and OS treatment samples were taken at 1.5 and 6 h, and expression profiling was analyzed as shown in the heat map (Figure 5) (Tables S9 and S12). *ZmAP2-2*, *ZmAP2-17*, *ZmERF58*, *ZmERF14*, *ZmDREB21,* and *ZmERF57* were overexpressed in the control condition but had shown no expression regulation under OS and wounding treatments.

*ZmAP2-5*, *ZmERF26,* and *ZmERF18* were only expressed in OS_1.5h treatment. Under OS_6h treatment *ZmDREB88*, *ZmAP2-1,* and *ZmDREB55* were overexpressed. After undergoing wounding treatment for 1.5_h, *ZmERF12*, *ZmDREB70*, *ZmDREB26*, *ZmDREB37*, *ZmDREB24*, *ZmDREB38,* and *ZmERF10* were highly expressed. *ZmRAV4* was highly expressed under W_1.5h treatment, and *ZmRAV4* and *ZmRAV2* were both overexpressed under OS_1.5h treatment. *Zmsoloist* was slightly regulated in response to wounding treatment. Under OF_8h treatment, *ZmERF18*, *ZmERF14*, *ZmERF32*, *ZmDREB86*, *ZmAP2-5*, *ZmERF57*, *Zmsoloist*, *ZmAP2-1,* and *ZmAP2-2* were overexpressed. Through the application of jasmonic acid (JA_8h), *ZmDREB42*, *ZmAP2-17*, *ZmDREB85*, *ZmRAV2*, *ZmDREB89*, *ZmDREB39*, *ZmERF56*, *ZmERF27,* and *ZmERF58* were overexpressed (Tables S8 and S11). Under biotic stresses, *Zmsoloist* was upregulated only under OF_8h treatment.

The Venn diagram concludes that there are 11 *ZmAP2*/*ERF* genes expressed solely under heat stress (Figure S5). The nine *ZmAP2*/*ERF* genes (*ERF53*, *ERF87*, *ERF98*, *ERF44*, *ERF39*, *ERF23*, *ERF18*, *DERB71,* and *DERB53*) were exclusively expressed under cold stress. The eight *ZmAP2*/*ERF* genes (*ZmDREB19*, *ZmAP2-2*, *ZmDREB74*, *ZmDREB45*, *ZmDREB60*, *DREB79*, *DREB52,* and *ERF95*) were uniquely expressed under salt stress. Five *ZmAP2*/*ERF* genes (*ZmERF12*, *ZmERF59*, *ZmERF19*, *ZmERF41*, *ZmERF105*, *Zmsoloist*, *ZmDREB88*, *ZmERF94*) were significantly regulated under OS_1.5h and the *ZmDREB85* gene was expressed specifically under OS_6h treatment. Six genes were significantly regulated under W_1.5h and only *ZmERF51* was uniquely expressed under treatment W_6h. The transcription abundance of ERF genes in maize under wounding, with or without oral secretions (OS) from *M. separata* is shown in (Figure S5, Table S12).

### 3.7. Relative Expression of ZmAP2/ERFs by qRT-PCR

**Figure 6:**
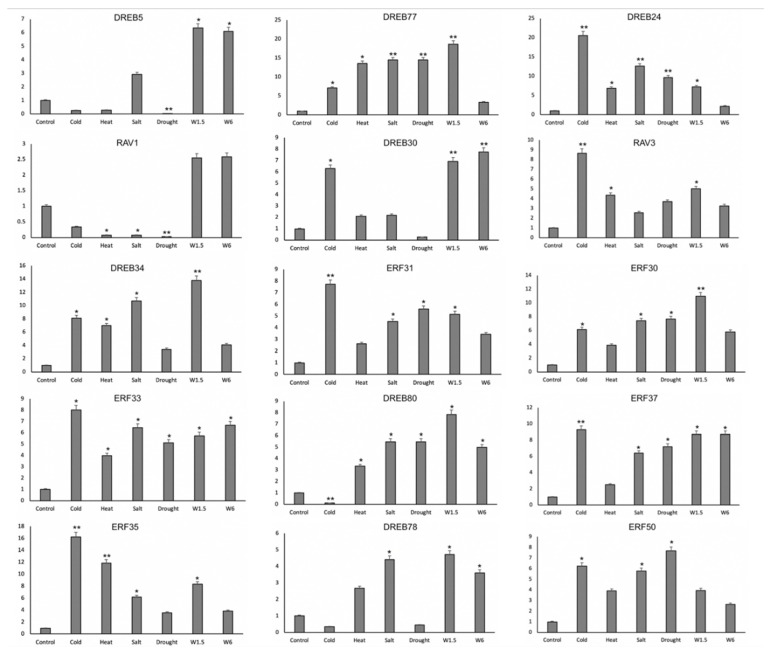
Expression profiling of fifteen selected *ZmAP2*/*ERFs* in response to cold, heat, salt, drought, and wounding stress (W1.5, W6) treatments. Data were normalized to *Actin2* and asterisks on bars indicate SD (* *p* < 0.05, ** *p* < 0.01).

For further confirmation that the *ZmAP2*/*ERFs’* expression is influenced by cold, salt, drought, heat, and wound 1.5- and 6-h stresses, we selected the overlap expression of biotic and abiotic stresses in 15 *ZmAP2*/*ERFs* genes for qRT-PCR (Figure 6). The *ZmDREB5* was highly responsive to wounding at 1.5 and 6 h. It showed no regulation under drought stress. *ZmDREB77* showed its regulation among all stresses but was expressed the least under the W6 treatment. *ZmDREB24* was expressed under all the conditions, but it was highly expressed under cold conditions. *ZmRAV1* was expressed the least among all the abiotic stresses, but it was highly expressed under W1.5 and W6 stress conditions. *ZmDREB30* was expressed under all stresses, and was highly expressed under cold, w1.5, and w6 treatments. *ZmRAV3* was significantly overexpressed under cold conditions. *ZmDREB34* was expressed under both biotic and abiotic stresses, and highly expressed under w1.5 treatment. *ZmERF31*, *ZmERF33*, *ZmERF37,* and *ZmERF35* were highly expressed under cold conditions. *ZmERF30*, *ZmDREB80,* and *ZmDREB78* were overexpressed under w1.5 treatment. *ZmERF50* was highly expressed under abiotic conditions as compared to biotic stress conditions, and significantly expressed under drought conditions.

## 4. Discussion

AP2/ERF is a ubiquitous family, composed of a large number of TFs with the ability to form complex stress-responsive networks [58]. It responds to biotic and abiotic stresses with erratic dynamic arrays: a number of them are stimulated rapidly and perpetually, however, some of them are induced by continued stress, which suggests that they may have a reciprocated effect on each other’s activity [58]. The AP2/ERF transcription family performs a substantial role in various developmental stages of the plant, i.e., it plays a significant role in the transcriptional regulation involved in complex growth, dynamical environmental stresses, seed germination, and floral development [58,59,60]. In the evolution of the Apetala2/Ethylene family, paralogous genes play a fundamental role [59]. These TFs act as a significant element in several plant mechanisms, as has been extensively studied in several plants, i.e., Arabidopsis [31], sorghum [60], rice [61], wheat [62], soybeans [33], grapes [37], castor beans [63], peaches [64], Hazel [65], *Arachis hypogaea* [66], and *Medicago truncatula* [67].

In the current study, the evolutionary processes of *ZmAP2/ERFs* were considered to find the variations in the members resulting in their novel functions. This family was extensively investigated, however, there is still diminutive knowledge about the maize *AP2/ERF* TF family. Due to the continuous updating of the maize genome database, a wide-range identification and characterization of the AP2/ERF transcription family remain to be further explicated in the latest version of the maize genome. In the current study, the *ZmAP2/ERF* family was investigated by using the V5 of the maize genome, resulting in the identification of 229 members with the AP2/ERF domain, varying as compared to the previous studies. Phylogenetic analysis and chromosomal localization were performed, which identified that maize followed a parallel distribution pattern of *ZmAP2/ERF* similar to that of other plant species [31,61]. Based on former classifications, the *ZmAP2/ERFs* were categorized into groups, i.e., DREB, AP2, ERF, RAV, and Soloist [68,69]. The *ZmAP2/ERF* enquiry led to the classification and identification of 229 members, with 105 ERF subfamily members, 27 AP2 subfamily members, 89 DREB subfamily members, 5 RAV subfamily members, and 1 soloist. Additionally, it is divided into the subfamilies DREBI-DREBIV and ERFV-ERFX. Each subfamily has distinct and prominent characteristics. The whole classification and distribution of *ZmAP2/ERFs* is comparable to that of other field crops [60,61,62].

Structural analysis of the ERF subfamily members revealed that 80% of them have no introns, whereas AP2 subfamily genes have 3–9 introns (Figure 2). The structural analysis of *ZmAP2/ERF*s revealed their similarity to *SbAP2/ERFs* [60]. The structural variation provides huge diversity in genome evolution. Generally, the ethylene-responsive factors are characterized by few introns, but among *ZmERFs,* the total number of introns is higher than in other plants. In total, 20 genes in *A. thaliana* [31] and 41 genes in *O. sativa* harbor introns [61]. It was identified that transposable elements (TE) are present in the introns of *ZmERFs*, which might have played a crucial role during whole-genome duplications and rearrangement events. These events might be involved in upsurging the number of genes and introns in *Z. mays*.

Among transcription factors, conserved motifs play a significant role in gene functioning. They are often associated with protein-protein interactions and different transcriptional activities. Motif analysis of *ZmAP2/ERF*s showed that the majority of *ZmAP2* confined Motif-1, Motif-2, Motif-3, and Motif-4 (Figure 2). Among the *ZmERF* subfamily, Motif-7, Motif-8, and Motif-10 were identified, concluding that they execute a central role in gene regulations. In the following study, the chromosomal location and segmental duplications analysis suggested that some *ZmAP2/ERFs* might have evolved by duplication and work as a major driving force for evolution. The promoter analysis revealed that *ZmAP2/ERFs* contained manifold ABRE, signifying that these elements are involved in ABA-dependent responses to salt in addition to water scarcity stresses.

In the above study, expression quantification of 229 *ZmAP2/ERFs* was detected. Apetala genes regulate crop yield and seed quality by controlling the development of embryonic cells and floral organs [70], whereas the ethylene-responsive factor controls the ET signaling network by binding to the promoter region (GCC box) of pathogenesis-related genes and affecting the fruit ripening [62,71]. Members of the RAV family play a principal role in the plant’s growth and developmental processes, i.e., leaf senescence [72]. *AtRAVs* and *AtAP2s* play vital roles in developmental processes, i.e., shoot and root apical meristem maintenance, flower initiation, etc. [59,60]. The results of this study showed that ethylene-responsive factors were significantly upregulated in silk and cob. The *DREB* factors regulate the root elongation and a significant upregulation of *ZmRAVs* was identified in pollen and meristem tissues.

Apetala and the ethylene-responsive factor family regulate several stresses, such as low temperature, drought, heat, and salt [61,62,63]. DREBs comprise several C-Repeat-Binding Factors (CBFs) that, together with transcription factor ICE, regulate the majority of the DRE comprising low-temperature responsive Arabidopsis genes [64,68]. Similarly, *OsDREB1s* and *OsDREB2s* in *O. sativa* [69,70], *TaDREB1* in wheat [71], and *HvDRF1* in barley contribute to stress tolerance [72]. Here, we examined the relative expression of 229 *ZmAP2/ERFs* under salt, cold, heat, drought, ABA, JA, oral secretion, OF, and wounding treatments.

Among them, *ZmAP2-1*, *ZmAP2-3*, *ZmAP2-16*, *ZmERF82*, *ZmERF59*, *ZmERF12*, *ZmERF7*, *ZmERF57,* and *ZmDREB86*, *ZmDREB85*, *ZmDREB80*, *ZmDREB77*, *ZmDREB16*, *ZmDREB26,* and *ZmDREB40* were highly expressed under salt, drought, and heat stress. A large number of genes increased their expression level under low-temperature stress. *ZmERF34*, *ZmERF42*, *ZmDREB30*, *ZmDREB31*, *ZmDREB22*, *ZmDREB29*, *ZmDREB83*, *ZmRAV2*, *ZmRAV3,* and *Zmsoloist* were highly expressed under cold stress. In response to biotic stresses, *ZmERF12* overexpressed after 1.5h of wounding stress. Members of AP2, ERF, and DREB overexpressed under treatment of oral secretions. Only *Zmsoloist* upregulated in response to OF_8h treatment.

AP2/ERFs affect the hormone-mediated stress responses, i.e., ABA and ET [62,63]. The subfamily of ethylene-responsive factors are the foremost downstream controlling elements of the ethylene signaling pathway [37,73,74,75]. Abscisic acid protects the plant against stresses by inducing stomatal closure, modifying root architecture, and synthesizing osmolytes [76,77]. *ZmDREB39* and *ZmDREB89* are upregulated in response to the application of both ABA and JA.

Jasmonic acid is a crucial signaling molecule for a plant’s growth and defense. It has a synergistic interaction with ethylene that initiates the defense-related genes in response to insect attacks and infection by different types of pathogens [78,79]. The factor ERF1 (At3g23240) acts as an integrator of jasmonic acid and ethylene signaling pathways in *A. thaliana* [80].

Jasmonic acid-inducible AP2/ERF-TFs, ORCA3 increases the accumulation of terpenoid indole alkanoids in C. roseus [81]. It initiates the strictosidine synthase (Str) expression by directly interacting with jasmonic acid and the biotic stress-responsive element (JERE) in its promoter regions [82]. The ORA59 (At1g06160) AP2/ERF transcription factor integrates jasmonic acid and ethylene signals to regulate the expression of the *PDF1.2* and *ChiB* genes [83]. The octadecanoid-responsive AP2/ERF-domain transcription factor 47 of *A. thaliana* is an *AP2/ERF* TFs, which controls JA biosynthesis and is induced by methyl jasmonic acid application [84]. *AtERF4* (At3g15210) negatively regulates the expression of PDF1.2 [79].

In this study, *ZmAP2-17*, *ZmRAV2*, *ZmERF56*, *ZmERF27*, *ZmERF58*, *ZmDREB42,* and *ZmDREB85* were upregulated while *ZmERF18* was downregulated in response to the Jasmonic acid treatment. Inclusively, the above findings provide an insight into the potential functional roles of the *ZmAP2/ERF* family and the candidate factors that will be used for the genetic improvement of maize.

## Data Availability

Available on NCBI.

## Supplementary Materials

The following supporting information can be downloaded at: https://www.mdpi.com/article/10.3390/genes14010194/s1. Supplementary Table S1: Basic information of AP2/ERF family; Supplementary Table S2: Paralogous pairs in Maize genome; Supplementary Table S3: Divergence time prediction between AP2/ERF family members; Supplementary Table S4: Orthologues of maize *AP2/ERF* genes; Supplementary Table S5: Transcriptome profiling of 23 tissues in maize; Supplementary Table S6: Transcriptome profiling of 79 tissues in maize; Supplementary Table S7: Transcript abundance of *ZmAP2/ERF* genes under stress environment; Supplementary Table S8: Transcript abundance of *ZmAP2/ERF* genes under JA treatment (OF, *Ostrinia furanacalis*, Asian corn borer); Supplementary Table S9: Transcript abundance under wounding with or without oral secretions from *Mythimna separata*; Supplementary Table S10: Differential expression under ABA, Cold, Heat and drought; Supplementary Table S11: Differential expression under JA treatment and insect feeding; Supplementary Table S12: Differential expression under wounding with or without oral secretions from *Mythimna separata;* Supplementary Table S13: Primers list of qRT-PCR. Supplementary Figure S1: LOGOS of conserved Motif Analysis, Supplementary Figure S2: Chromosomal location of *ZmAP2/ERF* genes; Supplementary Figure S3: Cis regulatory elements; Supplementary Figure S4: Heat map of AP2/ERF genes in different maize tissues; Supplementary Figure S5: Venn diagram under different abiotic stresses. References from Supplementary Materials: [45,85].
