# Supplementary figures and images for: Wide-Range Portrayal of AP2/ERF Transcription Factor Family in Maize (Zea mays L.) Development and Stress Responses
Source: Genes (Basel). 2023 Jan 11;14(1):194. doi: 10.3390/genes14010194 (PMC9859492; doi:10.3390/genes14010194)

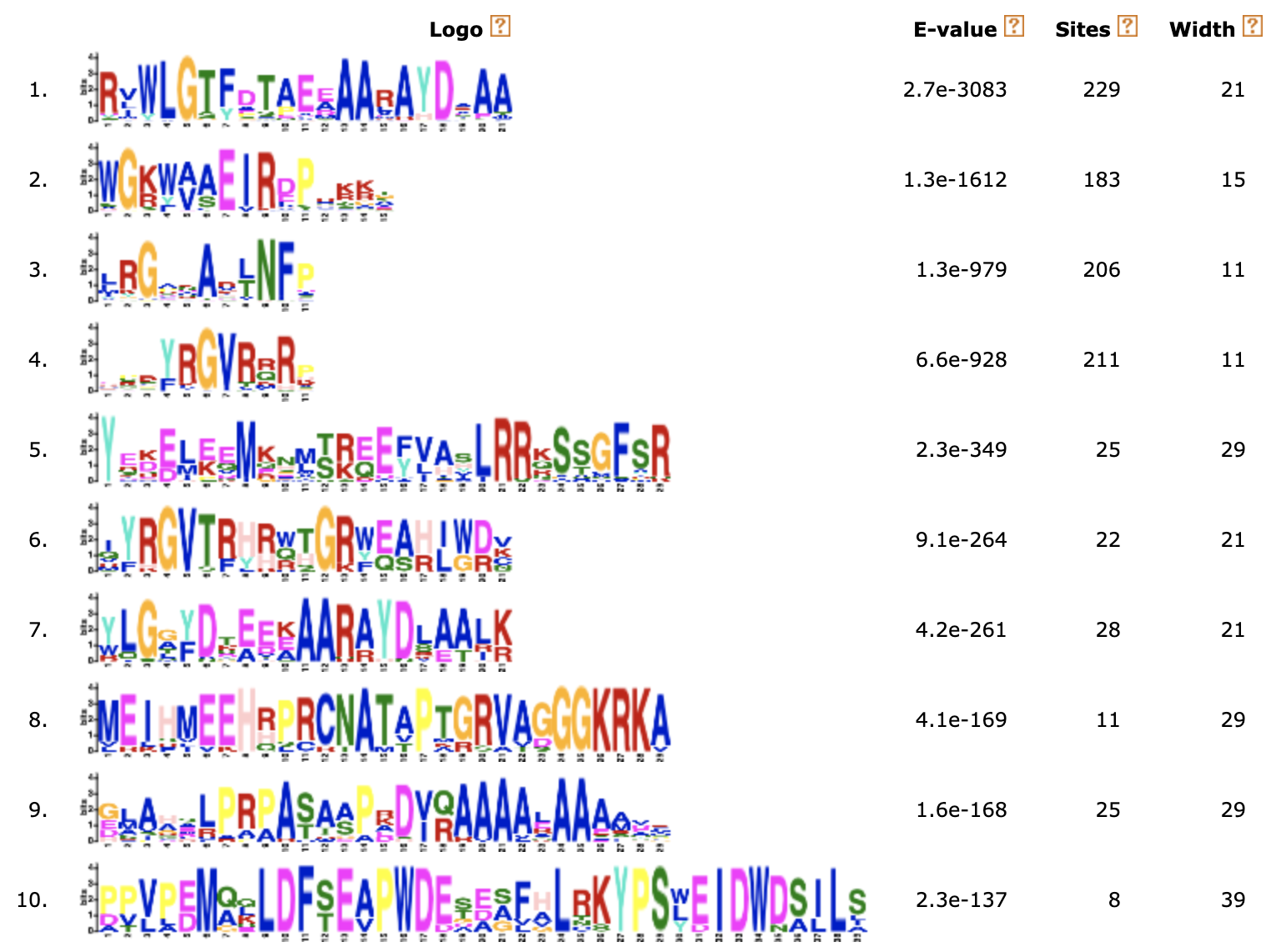

Supplement: Supplementary file 1 [file genes-14-00194-s001.zip › Supplement/Figure S1.tiff]

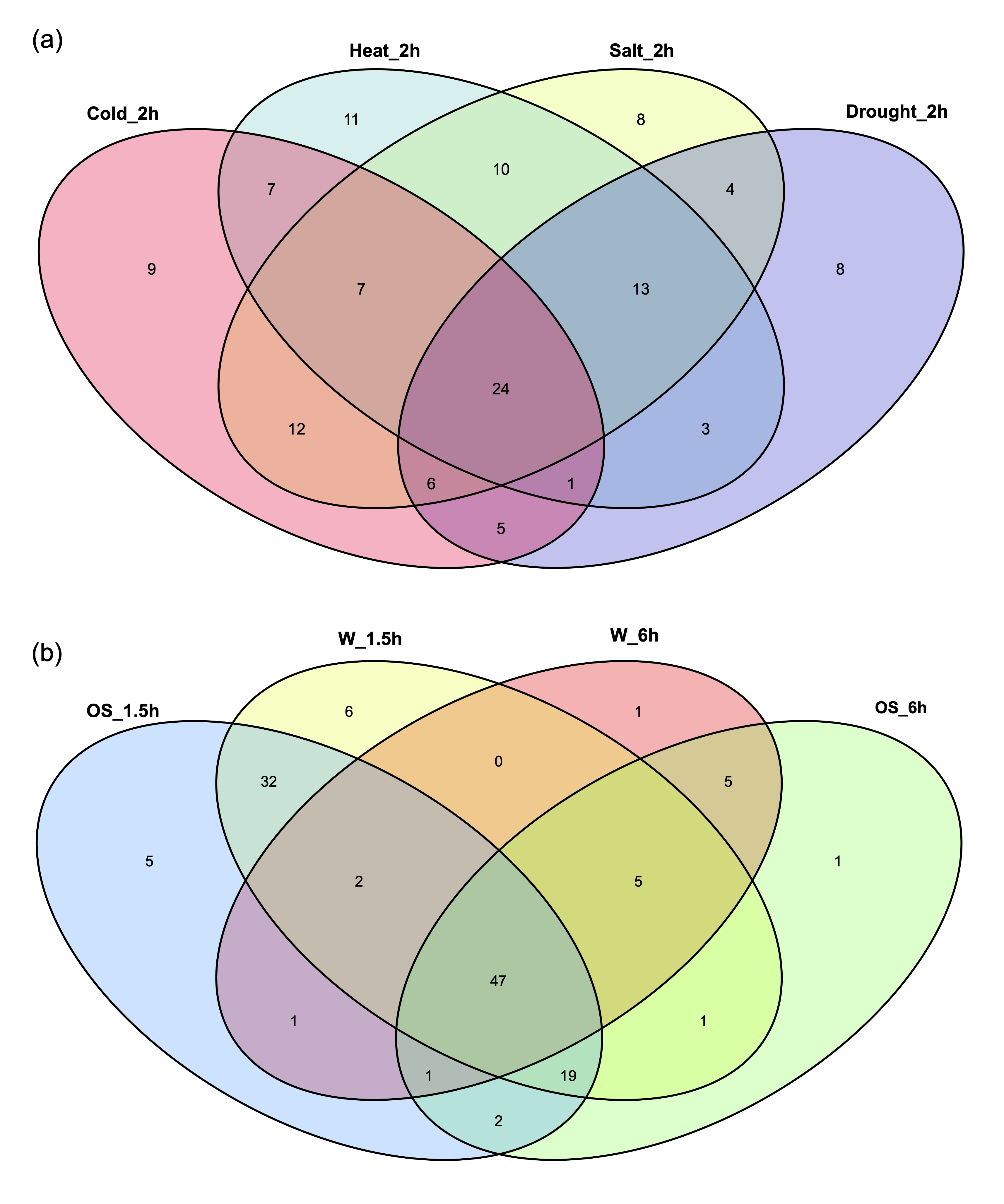

Supplement: Supplementary file 1 [file genes-14-00194-s001.zip › Supplement/Figure S5.tiff]

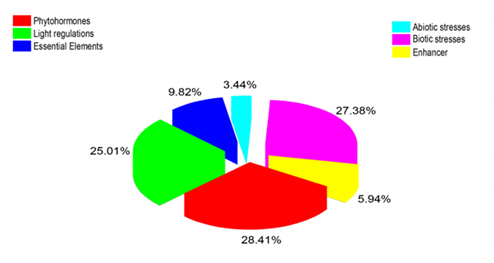

Supplement: Supplementary file 1 [file genes-14-00194-s001.zip › Supplement/Fiigure S3 B.tiff]
